# Using the Hands to Represent Objects in Space: Gesture as a Substrate for Signed Language Acquisition

**DOI:** 10.3389/fpsyg.2017.02007

**Published:** 2017-11-20

**Authors:** Vikki Janke, Chloë R. Marshall

**Affiliations:** ^1^English Language and Linguistics, University of Kent, Canterbury, United Kingdom; ^2^Department of Psychology and Human Development, UCL Institute of Education, London, United Kingdom

**Keywords:** gesture, locative expressions, classifier predicates, sign language, sign-naïve adults, adult second language acquisition

## Abstract

An ongoing issue of interest in second language research concerns what transfers from a speaker's first language to their second. For learners of a sign language, gesture is a potential substrate for transfer. Our study provides a novel test of gestural production by eliciting silent gesture from novices in a controlled environment. We focus on spatial relationships, which in sign languages are represented in a very iconic way using the hands, and which one might therefore predict to be easy for adult learners to acquire. However, a previous study by Marshall and Morgan (2015) revealed that this was only partly the case: in a task that required them to express the relative locations of objects, hearing adult learners of British Sign Language (BSL) could represent objects' locations and orientations correctly, but had difficulty selecting the correct handshapes to represent the objects themselves. If hearing adults are indeed drawing upon their gestural resources when learning sign languages, then their difficulties may have stemmed from their having in manual gesture only a limited repertoire of handshapes to draw upon, or, alternatively, from having too broad a repertoire. If the first hypothesis is correct, the challenge for learners is to extend their handshape repertoire, but if the second is correct, the challenge is instead to narrow down to the handshapes appropriate for that particular sign language. 30 sign-naïve hearing adults were tested on Marshall and Morgan's task. All used some handshapes that were different from those used by native BSL signers and learners, and the set of handshapes used by the group as a whole was larger than that employed by native signers and learners. Our findings suggest that a key challenge when learning to express locative relations might be reducing from a very large set of gestural resources, rather than supplementing a restricted one, in order to converge on the conventionalized classifier system that forms part of the grammar of the language being learned.

## Introduction

This study offers a fine-grained analysis of how adults with no knowledge of sign language (“sign-naïve adults”) begin to use their hands to represent objects in spatial relationships with other objects when required to do so without speech. The relevance of this research potentially extends beyond sign languages to linguistic theory more generally. Any theory of second language acquisition needs to be able to account for data on all languages, including languages in different modalities. An issue of considerable interest in second language acquisition research is what transfers from the speaker's first language to their second, in other words, identifying specific aspects of cross-linguistic influence (Jarvis and Pavlenko, 2008). Traditionally, one of the reasons that second language learners are thought to differ from native speakers is because their first language “leaks” into the new language. This is evident from foreign accents in pronunciation (Elliott, 2003), from word choice (Caroll, 1992; Janke and Kolokonte, 2015), and from sentence structure (Bardel and Falk, 2007), for example. However, while transfer has been extensively researched in the second language acquisition of spoken languages (e.g., Montrul, 2000; Siegel, 2003; Sharma, 2005; Gabriele, 2009; Gabriel and Kireva, 2014), and even to a certain extent with respect to manual co-speech gesture (Kellerman and Van Hoof, 2003; Gullberg, 2009), it has been largely neglected in studies of sign language acquisition (see Ortega, 2013 for a rare exception).

Having detailed knowledge of what learners start out with in terms of their gestural inventories before they begin to learn a sign language allows us to identify contenders for both negative and positive transfer. It is known, for example, that when asked to reproduce signs that resemble gestures that accompany speech, non-signers bring their gestural knowledge to bear on the task, the result of which can be a less accurately produced sign (see Chen Pichler and Koulidobrova, 2016, for a review of the literature). Conversely, Taub et al. (2008) have identified aspects of some sign novices' gestures, such as a natural ability to produce handshapes that closely resemble classifiers, which correlate positively with their later ability to engage in third-person discourse in American Sign Language. By providing a detailed picture of gesturers' manual resources, our study aims to enable connections to be established between what learners produce when acquiring sign and the resources they draw upon.

**Figure 1:**
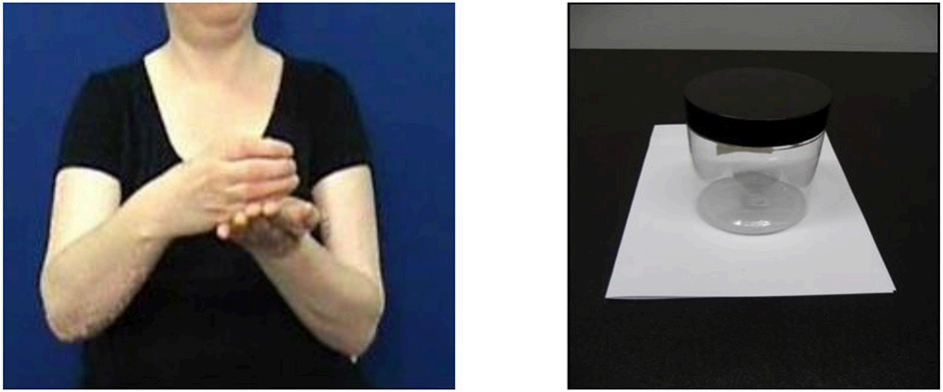
**(A)** CL-CURVED-OBJECT ON CL-FLAT-OBJECT. **(B)** Jar on sheet of paper.

Our focus is on how objects are represented in space. The visuo-spatial modality of sign languages allows signers to map spatial relationships, such as the relative locations of two or more objects, in a direct and very iconic way using their hands (what Brentari et al., 2012 term “hand-as-object” representations). For example, Figure 1A shows a signer of British Sign Language (BSL) expressing the spatial relationship between the two objects in Figure 1B. Her handshapes have the meaning of “object from the class of curved entities” (in this particular case, “jar”), and “object from the class of broad and flat entities' (i.e., “sheet of paper”). The orientation of her right hand shows that the jar is upright, rather than on its side or upside down. The location of the curved hand relative to the flat hand shows that the jar is on the paper, and not in any other spatial relationship. The handshapes that she is using to represent different classes of objects are termed “entity classifiers” (which Zwitserlood, 2012, terms “whole entity classifiers,” and which comprise both what Supalla, 1986, terms “static size and shape specifiers” and “semantic classifiers”; see Schembri, 2003, for a detailed classification of classifiers in sign languages). Importantly, different sign languages do not necessarily choose the same handshapes to represent the same classes of objects; entity classifiers differ cross-linguistically (Frishberg, 1975; Engberg-Pederson, 2010) and the set of handshapes used in classifier constructions is a subset of the handshape inventory for the language as a whole. A speaker would represent relationships such as those in Figure 1B very differently, using, depending on their language, lexemes such as prepositions, postpositions, circumpositions, locative case markers, or even positional and posture verbs (see Perniss et al., 2015, for a fuller discussion).

Speakers also make extensive use of their hands during speech (Kendon, 1980; McNeill, 1992), and these gestures complement their verbal communication in interesting ways. Indeed, such co-speech gestures can be similar in form to entity classifier constructions (see Table 1 in Marentette et al., 2016, for a useful summary of the similarities in form between signs and gestures). The frequency with which co-speech gestures occur and the types of gestures that are produced vary according to what a speaker is trying to convey (Kendon, 1980; McNeill, 1992). For example, a study by Lavergne and Kimura (1987) found that conversation involving spatial descriptions elicited double the number of gestures in adults compared to conversation unrelated to spatial descriptions. However, co-speech gestures vary not only in frequency but also in complexity. Representational gestures, for example, contrast with beat gestures (see McNeill, 1992; Kita, 2000; Alibali, 2005), where the former include a heterogeneous set (including handshapes, placement, and movement), which buttress the semantic content of an utterance, and the latter comprise a more basic and limited set of movements, which link to an utterance's rhythm.

The function of representational gestures means that they might provide the greatest insight into the rich gestural resources that sign-naïve speakers have at their disposal. However, co-speech gestures are rarely used to represent the complete semantic content of the utterances they accompany because this content is already encoded by the spoken words they are associated with. If we are to identify the full extent of the gestural resources that non-signers can draw upon, we need to provide a context in which the purpose of the handshape they produce is to fully represent a stimulus. In this respect, the term “dedicated gesture,” as introduced by Sandler (2012), is helpful; this describes the gesture recruited for a linguistic purpose, which gradually evolves, reflecting the move from pre-linguistic to linguistic articulation. A first step for researchers, then, toward achieving a closer evaluation of a speaker's gestural repertoire, is to increase the communicative function of the gesture. This can be achieved by studies of sign-naïve participants in which gestures replace speech altogether, which is the paradigm that we adopt here.

Our examination of how sign-naïve adults gesture visual stimuli that, in signers, elicit classifier constructions is motivated by the gestural properties of these constructions. Although there has long been debate over where classifier constructions are positioned on the gesture-sign continuum (Kendon, 1988; McNeill, 1992; see also chapters in Emmorey, 2003), there is growing recognition that they share many of the properties of gestures. For example, their movement, location, and orientation features are gradient rather than discrete. Furthermore, two-handed classifier constructions are not bound by the linguistic constraints that govern the formation of lexical signs (i.e., the symmetry and dominance conditions identified by Battison, 1978). Indeed, previous studies have shown that hearing adults who are asked to use gestures, but no speech, to describe how objects move in space will produce gestures which have some similarities to sign language classifier constructions (Singleton et al., 1993; Schembri et al., 2005; Brentari et al., 2012). In the current study, we monitor the way in which hearing adults with no knowledge of sign language (“sign-naïve adults”) use their hands to represent static spatial relationships between objects. In particular, we focus on the handshapes that they use to represent the objects that they are locating in space, what Perniss et al. (2015) term “entity representation.” Focusing on static, rather than moving, objects is expected to facilitate greater precision in our comparison of the handshapes of sign-naïve adults and of signers. The depiction of moving objects runs the risk of gesturers choosing to illustrate the path of the movement and not necessarily the object itself (see similar arguments for gestural ambiguity in Alahverdzhieva and Lascarides, 2010). Our focus on static objects avoids this potential confound. It also simplifies the task for participants, who need to concentrate on representing only three parameters, namely handshape, orientation and location, rather than handshape, orientation, and location plus movement.

Our study builds on work by Marshall and Morgan (2015), who investigated how accurately hearing adult learners of BSL used entity classifier constructions to describe changes in location and/or orientations of objects in pairs of pictures. The learners were all intermediate level students of BSL, who had been learning BSL for between 1 and 3 years. Although classifier constructions have been identified as an area of difficulty for hearing adult learners of sign languages (see Woll, 2012, and references therein), it is not clear which aspects of classifier constructions learners find challenging. Given the transparency of the mapping between the world and the “hand as object” in entity classifier constructions, and their potential gestural origins (e.g., Okrent, 2002; Liddell, 2003; Schembri et al., 2005), one might predict that they would be acquired easily and therefore produced accurately by this group of learners.

**Figure 2:**
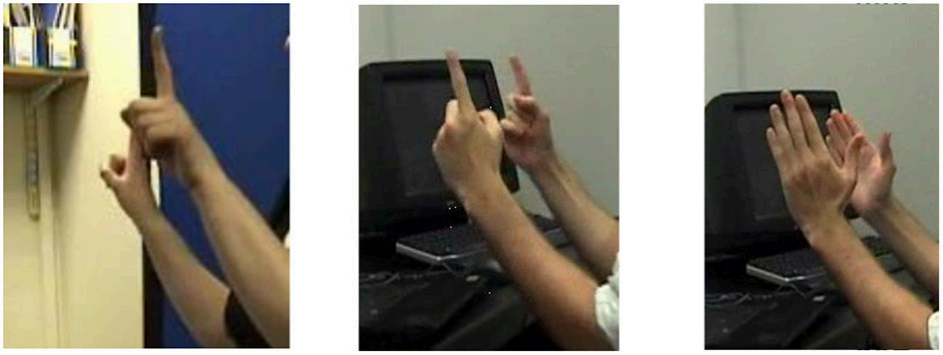
Over-differentiation in a BSL-learner's representation of two people standing next to one another. The native signer, in the photograph on the left, uses two upright index fingers to represent two people. The learner of sign also uses two index fingers in the photograph in the middle, but then changes to two flat hands in the photograph on the right.

In fact, the learners' productions did not match those of native signers very well (Marshall and Morgan, 2015). Although they did produce entity classifier constructions on approximately three quarters of the trials, on only one third of trials did they produce a handshape matching that used by native BSL signers. On the remainder of trials they used handshapes which were part of the inventory of BSL handshapes but which were not appropriate for the particular object being represented. Furthermore, learners used handshapes inconsistently, e.g., different handshapes for the same object within a trial. Figure 2 shows an example of this. The signers are describing a photograph of two people standing next to one another. The native signer, on the left, uses just the index finger to represent “person,” whereas the learner of BSL on the right uses first the index finger and then the flat hand. Marshall and Morgan (2015) also saw learners over-use the flat hand, which replaced other handshapes that native signers used. Thus, there were occasions when learners over-differentiated (i.e., used two or more handshapes to represent the same object) and other occasions when they under-differentiated (i.e., used one handshape to represent two or more classes of object). In contrast to the difficulty with selecting the correct handshape, learners were nearly always accurate at conveying the location and orientation of objects.

This relative difficulty for handshape over location and orientation was only present in Marshall and Morgan's production task, however. Participants were considerably more accurate in a forced-choice picture-selection task, in which they were presented with trials consisting of four pictures depicting objects in different spatial arrangements (Marshall and Morgan, 2015). Upon viewing the pictures, participants were shown a video-clip of a native signer producing a classifier construction that matched only one of the pictures. Participants succeeded in selecting the matching picture in nearly 90% of trials. Mean error rates for handshape, orientation, and location were all equally low. Importantly, participants did not make more errors for the handshape trials. For example, when presented with a video of a signer signing a classifier construction as in Figure 1A, and being shown pictures of different objects on a sheet of paper—jar, apple, coin, and pen—the signers were highly accurate in selecting the picture of the jar on the paper. In BSL, all those objects would be represented by different classifier handshapes.

Interestingly, when this comprehension task was carried out with a group of hearing adults who had no experience of sign language at all, a similar pattern of success was recorded (Marshall and Morgan, 2015). Although the sign-naïve adults were less accurate overall compared to the learners of sign, they, too, did not make more errors for handshape compared to location and orientation. The fact that they all performed significantly more accurately than chance suggests that it is not particularly challenging for people who have never seen a sign language before to map the shape of a signer's hands onto the correct referent when viewing entity classifier constructions. Note that this is quite unlike spoken second language acquisition, in which one would not expect a person to understand a non-cognate word in a new language on first exposure, thus rendering sign perception unique in this respect.

A question that immediately arises from Marshall and Morgan's (2015) production task is how sign-naïve adults might fare, given that learners found it so much harder to produce classifier constructions than to comprehend them. Because sign-naïve adults will, by definition, bring no sign language experience to the task, their spontaneous creations are likely to build upon their existing gestural abilities (as proposed by, *inter alia*, Taub et al., 2008; Brentari et al., 2012; Ortega, 2013). The learners of sign did have difficulty choosing the correct handshapes to represent the spatial arrangements of objects in the production task. Assuming that gesture is available as a substrate for learning a sign language, there are two alternative possible reasons for this difficulty. (1) Learners might have had few resources from gesture to draw upon, and in particular, a very limited repertoire of handshapes available to them to represent objects. This would imply that they were learning from scratch that the hand can take on different shapes to represent different objects. (2) Another possibility, however, is that learners did in fact have a substantial repertoire of handshapes at their disposal from gesture, and that the difficulties they exhibited in the production task stemmed from their needing to learn to select the appropriate, conventionalized, handshape for each object. On the basis of their participants' patterns, Marshall and Morgan (2015) stated that “gesture provides the substrate or the tools that learners recruit to sign with initially” but also that “this system needs to be reorganized for further development toward the system used by native signers” (p. 78). However, they did not discuss the alternatives (1) and (2), and because they provided no details of which handshapes were produced, it is not possible to tell whether the repertoire of handshapes that the learners were drawing on was smaller or larger than the repertoire of handshapes used by the native signers. This missing piece of the puzzle provides the motivation for the present study. By focusing on the handshapes produced by hearing adults with no experience of sign, we test these two alternatives by asking participants to describe the same pictures as Marshall and Morgan's (2015) participants, using silent gesture. Specifically, we examine how they exploit gesture when attempting to express spatial relationships with their hands. For comparison, we include a reanalysis of some of the data from learners of sign and native signers in Marshall and Morgan's (2015) study.

If the first alternative is correct—i.e., gesturers have only a limited repertoire of handshapes available to them to represent objects—we predict that participants will use only a limited set of handshapes as they complete the task. Indeed, they might make few attempts to create handshapes to represent objects at all, and might instead rely on pointing and enactments (i.e., positioning their whole body to locate the object) as they attempt to convey locative and distributional information about the objects in the pictures. If the second alternative is nearer the mark—i.e., gesturers have a substantial repertoire of handshapes at their disposal—we should see evidence of creativity with respect to handshapes used to represent objects, which will manifest in participants employing a wide range of handshapes that varies between and within participants. In line with the learners in the previous study, we would also expect to find instances of under- and over-differentiation, as well as some handshapes bearing strong similarities to those used by signers.

## Methods

### Participants

Thirty hearing British adults (12 male, 18 female) with a mean age of 32 years (*SD* 14; range 19–62) participated. This was an opportunity sample, drawn from undergraduate and graduate students at the universities where the authors work, and also drawn from the authors' acquaintances. Criteria for inclusion were that participants had never learned a sign language, were native speakers of English and reported no neurocognitive impairments. Confirmation of this information was collected via a brief language-history questionnaire, which was completed at the end of the testing session. Information regarding participants' additional languages was also collected from this questionnaire. 20 out of 30 participants had knowledge of one or more second languages, where knowledge was classified as at least an O-Level or GCSE (or its equivalent) in that language^1^. Participants were unaware of the specific research questions and hypotheses of the study—they were merely informed that the researchers were interested in how people use their hands to describe pictures.

In addition to the data from our sign-naïve participants, we reanalyzed for comparison some of the data from the learners of sign and the native signers in Marshall and Morgan's (2015) study. Data from these participants allows us to investigate what the conventionalized classifier system for a sign language (in this case, BSL) looks like, and to determine how close to that system a group of learners has moved. The learners of sign comprised 12 hearing adults (two male), with a mean age of 28 years (*SD* 6; range 22–44). They had been learning BSL for between 1 and 3 years. All of them had passed BSL Level 1 (beginner), eight had passed BSL Level 2 (intermediate), and three had begun classes at pre-level 3, in preparation for BSL Level 3 (advanced). Of the four adult native signers (one male), three were deaf and one was hearing.

### Procedure

Participants were seated at a laptop in a quiet room and informed that they would see two pictures in succession on the screen. Each picture featured two or more objects, whose location or orientation, or both, changed in the second picture (but the identity of the objects themselves did not change). Objects were chosen to elicit a range of handshapes, and included glasses, pens, books, toothbrushes, and toys such as human figures, airplanes, cars and motorbikes. Picture 1 was presented for 3 s, and then Picture 2 for 3 s, after which participants saw a large question mark on the screen. This was the cue for them to describe the pictures. Specifically, they were asked to explain, using only their hands and no voice, how the two pictures differed from each other, i.e., what had changed. This design had proved very successful at eliciting classifier constructions in Marshall and Morgan's (2015) study—the learners of BSL focused on describing just the relevant aspects of the scene, namely the relative locations and orientations of the objects depicted, rather than describing properties that were irrelevant for our purposes such as the color of the objects and their relative sizes.

The experimenter further told participants that it might help them to imagine that they were explaining the pictures to a profoundly deaf individual (similar to the instructions given by Schembri et al., 2005). Participants were not timed and could control the speed at which they progressed through the task. They were allowed to revisit a trial if they felt unsure about what they had just seen. Their responses were filmed, using a video camera mounted on a tripod, which was situated above and to the left of them if they were right-handed and above and to the right if they were left-handed.

### Stimuli

**Figure 3:**
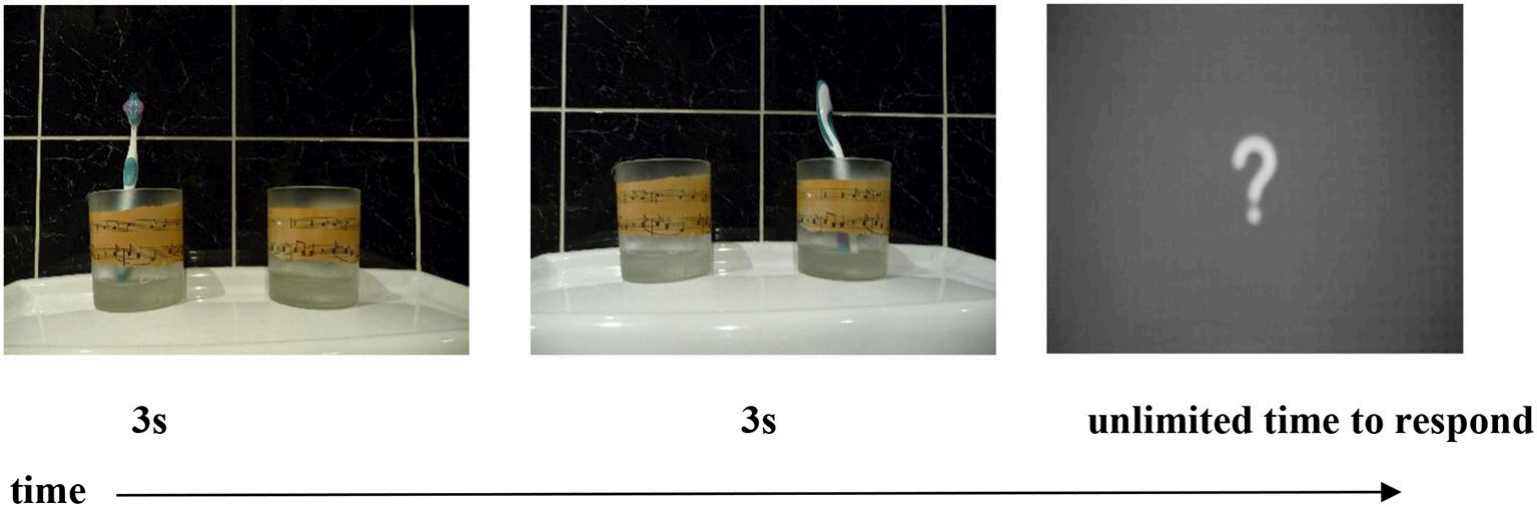
Example of a locative trial with a change in both orientation and location.

**Figure 4:**
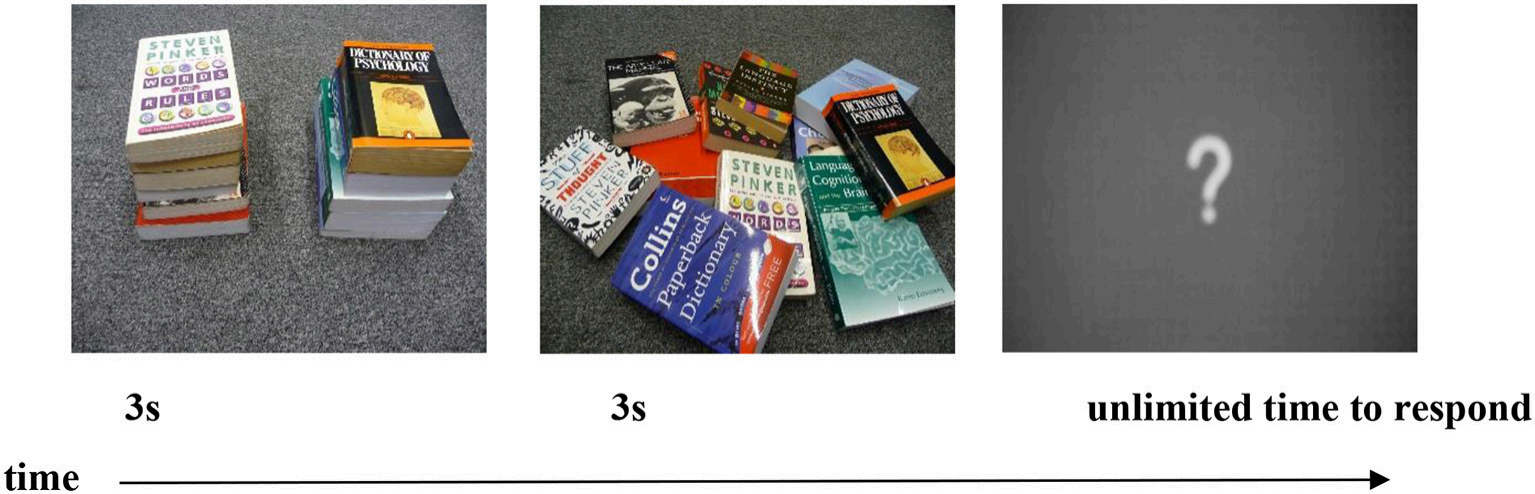
Example of a distributive trial with a change in distribution.

The stimuli were identical to those used in Marshall and Morgan's (2015) study. Two types of construction that elicit entity classifiers in BSL were included: locatives (i.e., X is at Y), and distributive plural forms. The locative construction included three conditions, namely, change of location, change of orientation, and change of both location and orientation (see Figure 3). There were 10 trials in each of these conditions. Of the 30 locative trials, three had just one object and six had three objects. The remaining 21 locative trials contained two objects. The distributive plural construction included one condition, namely change of distribution. This condition also contained 10 trials (see Figure 4), which resulted in a total of 40 trials for analysis. Two practice items trials were presented immediately after the instructions but not analyzed.

### Coding

In order to describe the pictures in the task (see Figures 3, 4), native signers divide their description into two parts. They first sign the lexical signs for the objects, and then produce a classifier predicate to give a spatial description of those objects. As reported by Schembri et al. (2005), Brentari et al. (2012), and Brentari et al. (2017), we expected gesturers either to do something similar, i.e., to create gestures to first describe the objects and then to show their relative locations, or alternatively just to describe the locations using gesture (given that their instructions were to “describe what has changed,” and only the location and/or orientation of the objects did change). Like Schembri et al. (2005) and Brentari et al. (2012, 2017), we coded just the spatial description part of the gestural sequence, and within that, only the handshapes that were used for that description, as for the current study we were not interested in the accuracy with which location/orientation were represented.

We anticipated that the set of handshapes produced by sign-naïve gesturers would not map exactly onto the set of conventionalized handshapes of BSL. Our coding system thus needed to capture not only those handshapes that did approximate those made by native signers but also those innovations for which there was no obvious BSL parallel. On this basis, handshapes were coded using the inventory and classification scheme devised for BSL as a whole by Brennan et al. (1984) which identifies five groups of handshapes according to finger joint configuration: fully closed, curved or bent, fingers together, fingers spread, and fingers extended from a closed fist. Every trial was coded by the two authors independently, who discussed any initial disagreements until agreement was reached.

Photographs of all the observed handshapes can be found in the appendix. They fell into four categories:

1. In the BSL inventory, and used by the native signers during the task.
2. In the BSL inventory, but not used by the native signers during the task.
3. Not in the BSL inventory, but variants of handshapes that are.
4. Not in the BSL inventory, and not falling straightforwardly into the five aforementioned handshape classes identified by Brennan et al. (Brennan et al., 1984). We labeled these “miscellaneous.”

**Figure 5:**
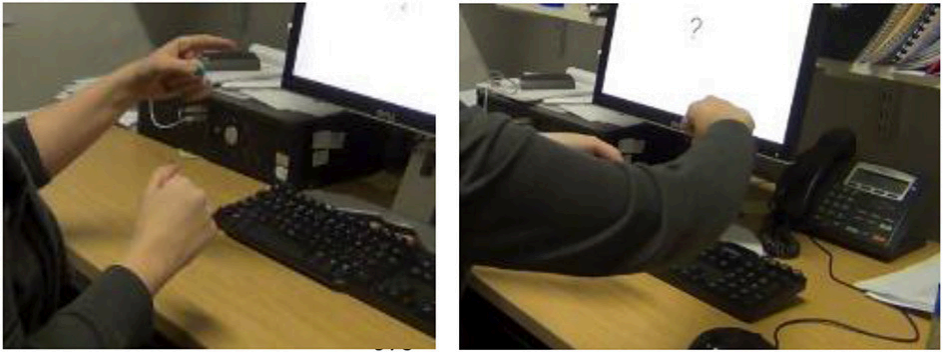
An illustration of the difference between use of an index finger placed horizontally to represent a motor bike (photograph on the left) and enactment of steering a motorbike (photograph on the right).

We also needed to distinguish between elicited productions that attempted to indicate the location of an object using the hands (analogous to the entity classifiers produced by signers doing this task) and those that indicated location by using the hands or whole body to mimic an action associated with the object or relied on the whole body to represent the object (we coded these as examples of enactment). Figure 5 illustrates the difference between these two possibilities. In response to the first picture, the participant places her index finger to indicate the position and orientation of a motorbike, which is facing her right. In the second, she relies solely on enactment to depict the change in orientation of the bike, which now faces her left. She uses her hands to represent holding the bike's handlebars and steering to the left.

Finally, we also coded instances in which a participant pointed to locate an object in space.

### Ethical approval

This study was carried out in accordance with the recommendations of University of Kent's Research Ethics Advisory Group for Human Participants. All participants gave written informed consent in accordance with the Declaration of Helsinki. The protocol was approved by the University of Kent's Research Ethics Advisory Group for Human Participants.

## Results

In order to investigate the range of handshapes exploited by sign-naive gesturers in their spatial descriptions of objects, we carried out three analyses. The first simply calculated how frequently gesturers use their hands to represent the relative location and/or orientation of objects in space. The second focused on the inventory of handshapes that gesturers draw upon in their spatial descriptions, and how that inventory compares to that of native signers and learners of sign. In the third, we investigated whether gesturers consistently produce the same handshape to represent the same object.

### The proportion of trials for which gesturers used their hands to represent at least one of the objects in their spatial descriptions of the elicitation stimuli

**Figure 6:**
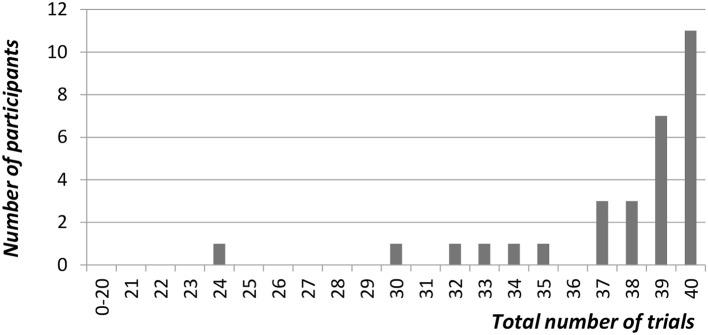
The total number of trials for which participants used their hands to represent an object at least once.

We first calculated the proportion of trials for which gesturers made at least one attempt to use “hand-as-object.” The total number of trials was 40. The data were negatively skewed, as shown in Figure 6 below: the group mean was 37.5 (i.e., 93.8%), the median 39 and the mode 40.

Having found that hands were used to represent objects in 93.8% of trials, we then looked to see how participants were responding in the remaining few trials, and found them to be distributed between instances of enactments (2.1%), pointing (3.2%), and trials in which participants failed to attempt to represent the objects at all (0.8%).

Our interpretation of these data is that gesturers readily use their hands to represent objects when describing the relative locations and/or orientations of those objects, as signers do. However, this does not mean that they are doing the same thing with their hands as native signers. Although they use their hands to represent objects, do they create handshapes that are comparable to those of native signers? And how similar are their handshapes to those of hearing adults who are learning sign? We investigate these questions in the next section by comparing the number of handshapes produced by gesturers with those of the native signers and learners of BSL in Marshall and Morgan's (2015) study.

### The inventory of handshapes that sign-naïve gesturers drew upon, and how that inventory compares to native signers/learners of sign

We first compared the handshapes produced by gesturers to those produced by the group of native signers. The number of handshapes used by gesturers on the task ranged from 4 to 19 (Mean = 12.47, *SD* = 2.99). From this set, the number of handshapes that overlapped with those used by the group of native signers ranged from 2 to 11 (Mean = 7.10, *SD* = 1.99), while the number of handshapes that were different from those used by native signers also ranged from 2 to 11 (Mean = 5.37, *SD* = 2.04). Therefore, the gesturers each produced handshapes that were the same as those used by signers and likewise each produced handshapes that were different to those used by signers, despite there being a wide range in the number of handshapes that each gesturer used in the task. The only handshape common to all 30 gesturers was the flat handshape (see photograph 16 in the appendix).

**Figure 7:**
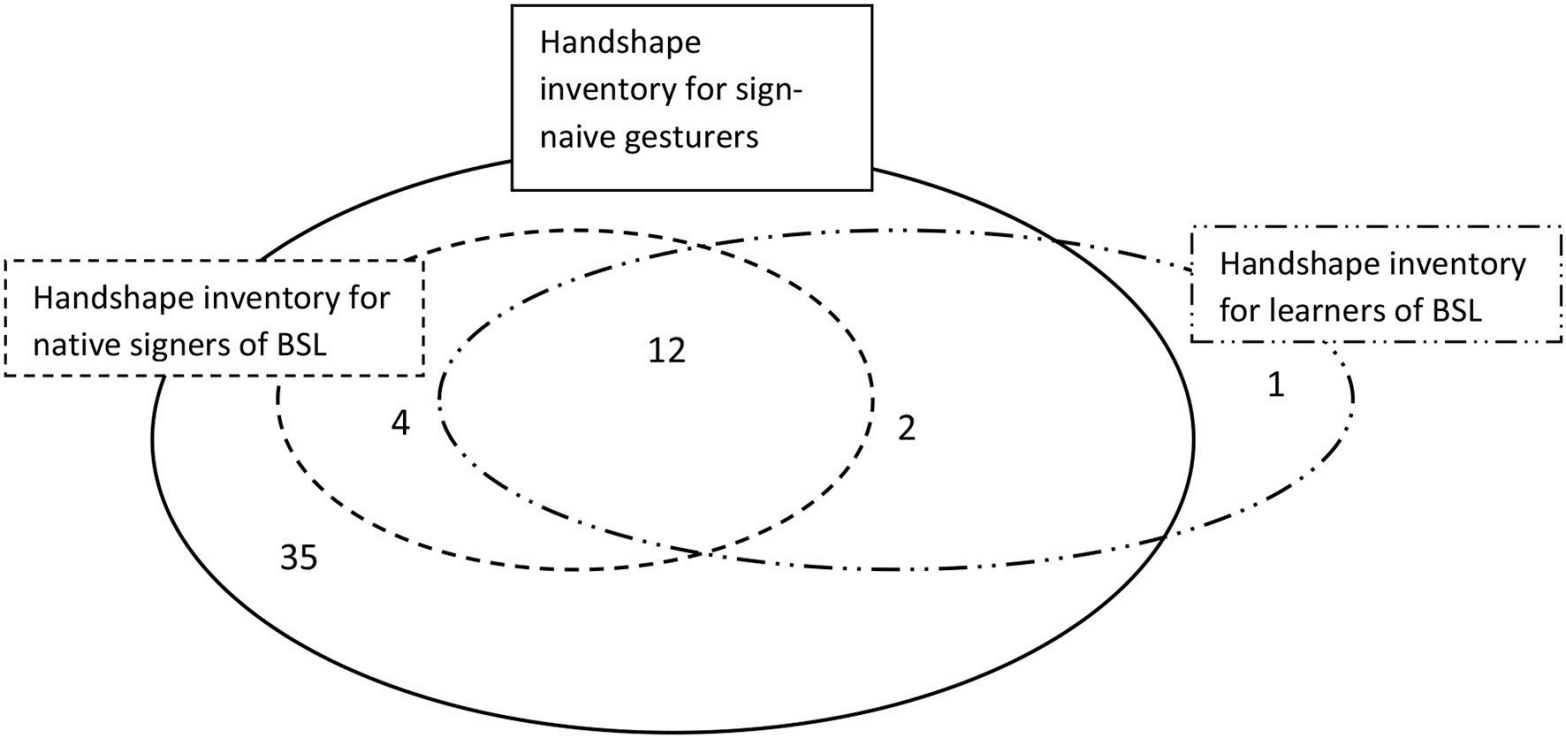
The overlapping entity classifier handshape inventories of sign-naïve gesturers, learners of BSL, and native signers. Numbers represent the number of handshapes at the intersections of the different inventories (e.g., 12 is the number of handshapes that occurs in the inventories of all three participant groups: native signers, learners of BSL and sign-naïve gesturers).

Considering the sign-naïve group as a whole, the number of distinct handshapes produced by the sign-naive gesturers in the task was 53. These handshapes are all shown in the appendix. A reanalysis of Marshall and Morgan's (2015) data revealed that the number of handshapes employed by the native signers and the learners of sign was much smaller, namely 16 for the native signers and 15 for the learners, and those two sets overlapped almost exactly. Gesturers therefore had a very wide selection of handshapes available to them, a near superset of what native signers and learners of sign used. This situation is illustrated schematically in Figure 7.

From Figure 7, we can see that the sign-naïve gesturers generated all the handshapes that the native signers did. In addition, they created a number of handshapes not found in BSL, or they employed BSL handshapes in different ways to signers. We discuss some relevant observations below, starting with those handshapes not found in BSL.

**Figure 8:**
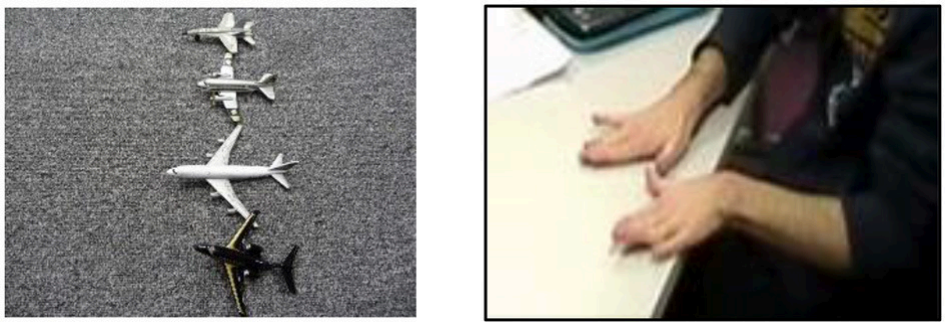
This participant is using a novel handshape to gesture planes lying side by side.

Firstly, four participants independently converged on a handshape to represent “plane” that does not fit neatly into Brennan et al.'s (1984) classification scheme for BSL, and which to our knowledge does not occur in the inventory of any sign language (although it should be noted that there is no exhaustive list of all the handshapes in existence in all the world's sign languages, so our unawareness of its existence does not imply that it does not exist). This handshape—where the index, middle and ring finger were extended horizontally together, whilst the thumb and little finger were projected out either side—is illustrated in Figure 8, and in photograph 51 in the appendix. Interestingly, Schembri (2001, Table 5.46, p. 229) reported five instances of Australian sign-naïve gesturers using this same handshape when they were describing the motion of an airplane, and noted that none of the Australian and Taiwanese Sign Language users who also took part in the study used it.

**Figure 9:**
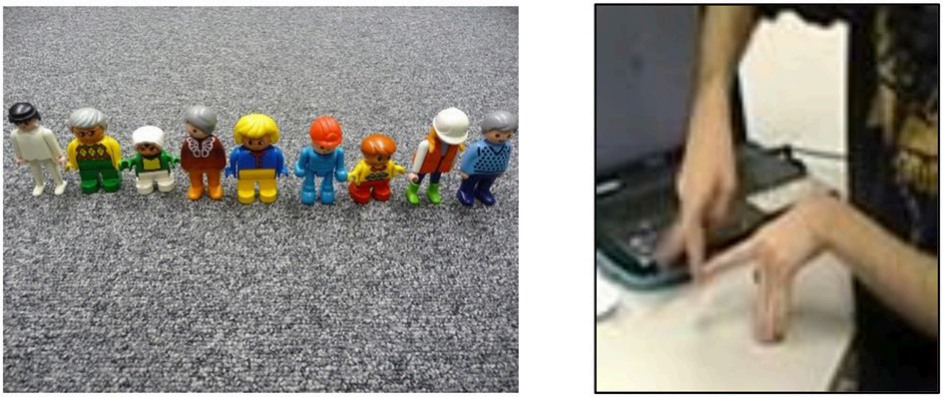
This participant is using a novel handshape to gesture people standing in a row, using the index finger to represent the direction in which the people are facing and the middle, ring and baby fingers the legs.

One of our participants created an unusual handshape to represent people, which he relied on consistently throughout all trials. His middle, ring and baby fingers were held vertically, pointing down, in what appears to be an attempt to represent a person's legs, whereas his index finger pointed horizontally in the direction in which the person was facing. By exploiting the index finger to encode the direction in which the figure was looking, the participant managed to convey several aspects of his target simultaneously with one handshape. Figure 9 and photographs 48 and 49 in the appendix demonstrate this handshape.

**Figure 10:**
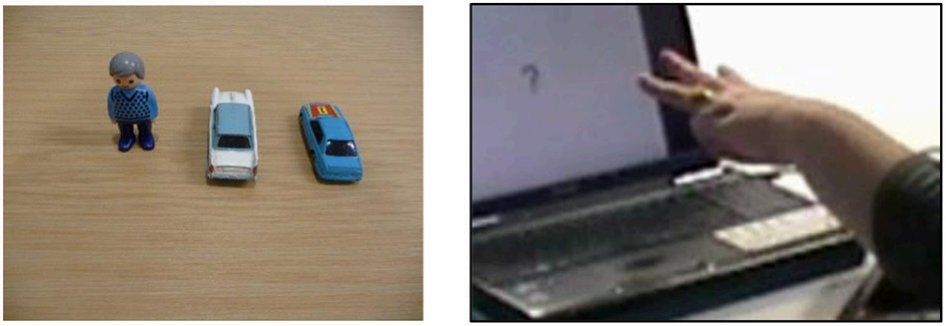
This participant is using the three handshape to represent three objects located next to one another.

Another interesting observation is that even when gesturers produced a handshape that is part of the BSL handshape inventory, they sometimes used it in different ways to the native signers of BSL and the BSL learners. And yet, what the gesturers were doing with this handshape has parallels in another sign language. For example, five participants made varied attempts at representing something akin to the “next to” construction found in Turkish Sign Language (but not in BSL). These attempts materialized when participants were faced with photographs in which more than two objects were shown and so had to overcome the problem of having too few hands to depict all the objects simultaneously. Native signers of BSL and learners of BSL overcame this problem by representing objects sequentially rather than trying to represent them all at the same time. Some of our participants, however, constructed a simultaneous representation. One participant, for example, formed a handshape with the index, middle and ring fingers extended (palm down), using the three extended fingers to represent a row consisting of two cars and a person (see Figure 10).

**Figure 11:**
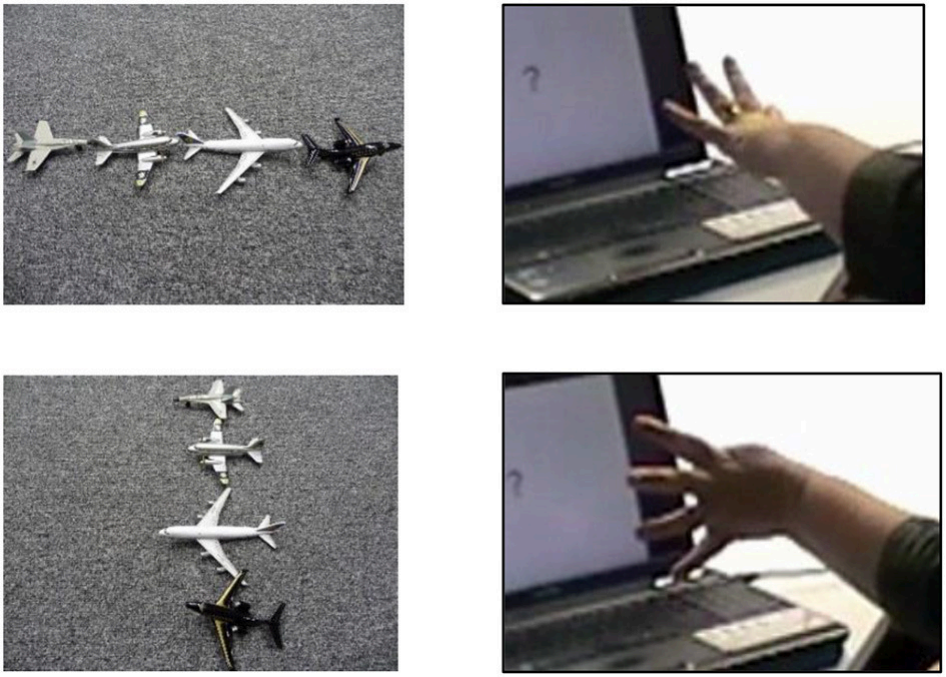
This participant is describing a row of four planes with the four handshape.

This same participant produced the four handshape when needing to depict four planes, changing the orientation of her hand as shown in Figure 11.

These attempts at expressing the “next to” relation are similar to locative predicates that are licit in Turkish Sign Language (Özyürek et al., 2010; Perniss et al., 2015). Özyürek et al. (2010), for example, reported an experiment in which six (deaf) Turkish signers were required to describe objects depicted in a photograph in sign to another (deaf) Turkish signer who could not see that photograph. Although the signers used locative predicates far less frequently than classifiers to represent spatial relations, all six of them relied on the locative predicate, “next to” at some point when describing a photograph in which the number of objects was greater than two. The horizontal three handshape, for example, was adopted to depict three plates in a row (see Perniss et al., 2015, Figure 4, p. 621), and the four handshape was produced to illustrate four cups in a row.

In both these examples from Turkish Sign Language, however, the multiple objects that these native signers needed to represent were always of the same type. They were not required to describe the position of several differently shaped objects. For this reason, it is not clear whether the locative predicate would be a licit means of representing different objects (i.e., cars and person, Figure 10) next to each other or whether the locative predicate would be restricted to depicting objects of the same type. If the “next to” relation in Turkish Sign Language is restricted in this way, some of our gesturers are showing a more flexible strategy than is permitted in that language. What is interesting for our purposes, however, is that five of our participants came up with this gestural strategy spontaneously when presented with this unforeseen challenge.

**Figure 12:**
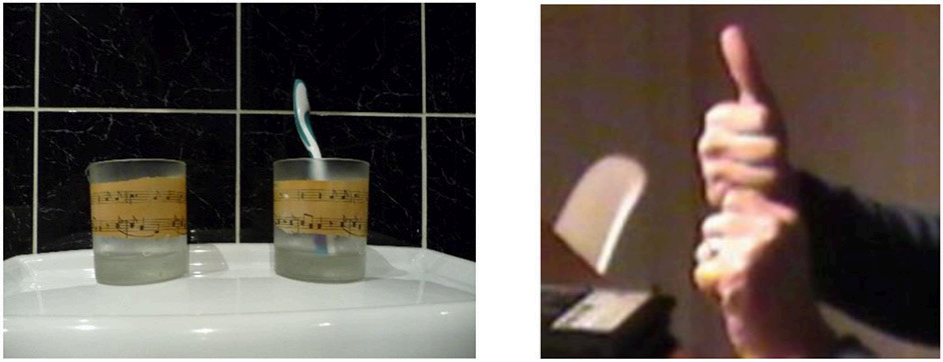
This participant is using two fists to represent the cups, with an extended thumb to represent the toothbrush.

Motivated by the same challenge, namely that of depicting more than two objects, some participants converged on other handshapes when attempting to represent these objects simultaneously. Some of the shapes they created can be found in the BSL inventory, albeit with a different function. In one trial, for example, participants were presented with pictures of two cups, one of which held a toothbrush. Three participants employed the handshape illustrated in Figure 12 below, and in photograph 1 of the appendix, to convey the cup with the toothbrush poking out of it, where their fist depicted the cup and their thumb represented the protruding toothbrush.

In another trial, participants were faced with two pens laying either side of a notepad. One participant made a point shape for one pen but produced a flat hand with her pinkie finger stretched out to the side to capture the second pen (see photograph 53 in the appendix). There were several more examples of these creative efforts to deploy the hands to illustrate three or more objects simultaneously, seemingly to avoid having to represent them sequentially (such as the handshape in photograph 32 of the appendix, with the index and middle fingers crossed, which some gesturers used to represent two crossed pens so that their other hand was free to represent a sheet of paper).

Despite the large set of handshapes used by the group of gesturers, the majority of them—37 out of 53 handshapes—are licit in the phonological inventory of BSL as a whole (see the appendix), although signers would not use them all in entity classifier constructions. Of the remaining 16 handshapes, seven appeared to be variants of handshapes in the BSL inventory, and four of those occurred only once in our data. The final 9 handshapes do not fit neatly into the classification scheme for BSL handshapes (i.e., the ones labeled “miscellaneous” in the appendix), but only one of those handshapes was used by more than one person (as shown in Figure 8). The overall picture with respect to the handshapes used by gesturers can therefore be summarized as follows: (1) At a group level, our sign-naïve gesturers draw on an inventory which is a superset of that used by the native signers in entity classifier constructions, but they rarely produce handshapes that are unlike those found in the entire handshape inventories of BSL and other sign languages; (2) At an individual level, no gesturer draws on exactly the same inventory of handshapes as the native signers of BSL would use in classifier constructions, and they sometimes use handshapes in different ways to the native signers (e.g., by representing two or more objects on the same hand).

We now turn to our third comparison, which considers how consistently participants employed the same handshape to represent a particular object when it occurred several times during the course of the 40-trial experiment.

### Handshape consistency across trials

The native signers in Marshall and Morgan's (2015) study proved remarkably consistent in using the same handshape to represent a particular object when that object occurred on multiple occasions. The learners in their study were not so consistent, however, and the sign-naïve gesturers in our current study are even less so. We examined the three groups' responses for six objects that are represented by different handshapes in BSL, namely car (photo 16 in the Appendix), plane (photo 40), pen (photo 26), book (photo 16), person (photo 26), and glass (photo 7). Each of these objects occurred a minimum of five, and maximum of six, times throughout the task, enabling us to track inter-trial consistency and to compare it across groups. As evident from Table 1, which displays the range and central tendency measures of the number of different handshapes used for a particular object, there was most variability in the sign-naïve gesturers, less variability in the learners and least of all in the native-signers.

**Table 1:** Inter-trial consistency of handshapes chosen for six objects by three groups: sign-naïve gesturers, learners of BSL, and native signers^a^.

	**Sign-naïve gesturers (*****n*** = **30)**				**Learners of BSL (*****n*** = **11)**				**Native signers (*****n*** = **4)**			
	**Number of handshapes**				**Number of handshapes**				**Number of handshapes**			
	**Range**	**Mean**	**Median**	**Mode**	**Range**	**Mean**	**Median**	**Mode**	**Range**	**Mean**	**Median**	**Mode**
Car^b^	1–9	3.1	3	2	0–3	1.73	2	1	1–2	1.33	1	1
Plane	1–6	2.4	2	2	1–3	1.64	1	1	1–1	1	1	1
Pen	1–6	3.5	3	3	1–3	1.73	2	1	1–1	1	1	1
Book	1–6	2.4	2	2	1–2	1.27	1	1	1–1	1	1	1
Person	1–5	2.9	3	4	0–5	1.18	1	1	1–2	1.33	1	1
Glass	1–6	3.2	3	4	1–3	1.45	1	1	1–2	1.33	1	1

a *Data from the latter two groups originate from Marshall and Morgan (2015)*.

b *Aside from car, which occurred six times, there were five occurrences of each object*.

## Discussion

This study investigated the handshapes that hearing adults with no knowledge of sign language create when asked to use just their hands, and no voice, to describe pairs of pictures where the relative location and/or orientation of one or more objects changes. We compared these handshapes with the classifier constructions produced by native signers and by learners of BSL. Hypothesizing that manual gesture is a substrate for sign language learning, we envisaged two potential scenarios: either sign-naive gesturers would not readily exploit their hands to represent objects when describing their spatial arrangement (and if they did, would produce only a limited set of handshapes relative to signers), or, alternatively, they would employ a much wider set of handshapes than signers. In each instance, there is a gap between the handshapes produced by the silent gesturer (which are not linguistically constrained, e.g., Özçaliskan et al., 2016) and handshapes that a native signer would produce. However, the alternatives diverge in terms of the nature of this gap. Thus, the hypotheses have different implications for the task of the sign language learner.

Three main findings emerged from this study. First, for the vast majority of trials, sign-naive gesturers used their hands to represent objects and to give spatial descriptions that looked similar in many ways to the entity classifier constructions produced by signers. Second, the group as a whole drew upon an inventory which is a superset of that of those used in the classifier constructions of native signers, and yet they rarely produced handshapes that are unlike those found in BSL as a whole and other sign languages. When looking at individual gesturers, we found that each used some handshapes that were identical to those produced by native signers and some that were not used by native signers, and they sometimes used handshapes in different ways to the native signers (e.g., by representing two or more objects using a single hand). Third, whereas individual native signers consistently used specific handshapes to represent particular objects, as did learners of sign, the sign-naive gesturers were much less consistent, with the majority employing a variety of different handshapes to represent the same object across the trials in the task.

We argue that these findings are all consistent with the following interpretation, namely that the challenge for hearing adults when learning to use classifier constructions in a sign language is in learning to select the appropriate, conventionalized, handshapes from a large repertoire of possible handshapes that are available to them by virtue of the large articulatory range of the hands. In other words, the task for learners of sign is not to learn how to represent objects using their hands, but rather to narrow down the set of handshapes that they have potentially available to them to the set of classifier handshapes that is grammatical in the sign language they are learning, and to select from that set accurately and consistently. In the remainder of this section we discuss each of the three findings in turn and motivate our interpretation of the data.

Turning first to the frequency with which gesturers employed their hands to represent objects, we found that the proportion of trials for which gesturers used their hands to represent at least one object on each trial was 94%, which is higher than what Marshall and Morgan (2015) reported for learners of sign (around 75%). We need to be cautious in comparing these figures directly because the task instructions for the two groups were not the same^2^ and so presumably the two sets of participants approached the task differently. Nevertheless, our findings show that learners do not lack the ability to represent objects with their hands, so this cannot be the reason that they find classifier constructions difficult. Gesturers did sometimes draw on other gestural possibilities, such as pointing and enactment (i.e., positioning their whole body to locate the object), but they did so only rarely. Instead, participants appeared to find it quite natural to exploit their hands to represent objects.

On many trials, gesturers produced handshapes that were not used by the native signers of BSL in Marshall and Morgan's (2015) study, but few handshapes fell outside the repertoire of BSL handshapes, which is consistent with the notion that some handshapes are physiologically harder to produce than others and are hence less likely to occur (Mandel, 1981; Ann, 2006). Interestingly, some gesturers produced handshapes that form part of the inventory of BSL classifier handshapes but they deployed them in ways more akin to other sign languages. For example, some of our gesturers used the handshapes with three and four fingers extended to represent multiple objects lying side by side in a way that is similar to how the “next to” handshape is used in Turkish Sign Language (and possibly in other sign languages too). We also found that objects were not represented consistently, and that speakers often adopted more than one handshape to represent the same object across (and even within) trials. This finding is consistent with the studies of Schembri (2001) and Schembri et al. (2005) for Auslan (Australian Sign Language, which is historically closely related to BSL). Like us, Schembri (2001) and Schembri et al. (2005) investigated sign-naïve participants gesturing silently, although the classifier constructions that they elicited required movement—they were not static like ours. In both studies it was noted that gesturers produced a greater number of handshapes than signers did to represent each category of object. This similarity between Schembri's findings and ours suggests that our task is tapping into resources that are not restricted to the participants who undertook our particular study. Furthermore, assuming that hearing adults draw on the resources available to them in manual gesture when they start learning a sign language, our findings and those of Schembri and his colleagues are consistent with the interpretation that what is challenging for learners of sign is to narrow down the many options provided to them in gesture in order to converge on the narrower conventionalized system of the particular sign language that they are learning.

There are some limitations of our study. The first is an obvious one: sample size. As can be seen in the appendix, only two thirds of handshapes were produced by more than one participant, meaning that the gestural handshape inventory that we compiled would have been different if we had recruited different participants, and likely larger if we had recruited a larger sample. The inventory presented in the appendix is therefore to a certain extent an artifact of sampling, and unlikely to be replicated exactly. Nevertheless, our sample size (*N* = 30) compares well with other similar studies (*N* = 22 in Brentari et al., 2017; *N* = 25 in Schembri et al., 2005), and the study's substantive findings are surely likely to remain if the sample size were bigger.

Secondly, the task was not embedded into a communicative context. A future study might create a paradigm in which the elicited gestures are integrated into a communicative event; such stimuli might give rise to a different pattern of results. A further interesting avenue to explore in subsequent work would be to elicit gestures together with speech for the same items, in order to better understand whether some of the more idiosyncratic handshapes we found are also present in the same gesturers' co-speech gestural repertoire. We had included in our instructions to participants that “it might help you to imagine that you are explaining the pictures to a profoundly deaf individual,” an instruction that has not always been included in previous studies of silent gesture (although it was in the study by Schembri et al., 2005). This instruction might have encouraged participants to create more elaborate handshapes in an attempt to provide greater specificity than would have been the case otherwise. Indeed, there were many examples of participants drawing on iconicity to create handshapes that resembled the form of the objects being depicted much more than the conventionalized BSL handshapes do (recall, for example, Figure 9), suggesting that it was important for them to recreate the appearance of objects as accurately as they could. The possibility that our instructions did encourage such elaborate handshapes is not necessarily problematic for our study though—most people who choose to learn a sign language such as BSL do so with the aim of communicating with deaf people, and the learners from Marshall and Morgan's (2015) study (whose data were reanalyzed in the current paper) were presumably approaching this task with communication with deaf people in mind. So although the large number of handshapes elicited in this study may be due to the particular nature of the task itself, and the same participants might produce fewer handshapes in their spontaneous co-speech gesture, we would still argue that we are tapping into speaker's gestural resources. Our particular task has allowed us to uncover just how varied those resources are.

In future work the process of how hearing adults learn a grammatically-constrained classifier system needs to be investigated. So far in our research we have studied gesturers who have never previously been exposed to BSL (current paper) and learners of BSL at a point in time corresponding to between 1 and 3 years of BSL learning (Marshall and Morgan, 2015). We have drawn some inferences about the learning process but, not having studied it directly, we do not know what this process looks like, and, in particular, how the set of classifier handshapes that is grammatical in the language being learned is actually acquired in the early stages of BSL learning. Crucial to such a study would be a close monitoring of the instruction that BSL students receive—in particular, the extent to which classifiers are explicitly taught. One consideration is the relatively low frequency with which classifier constructions occur in spontaneous conversation, as reported in Fenlon et al. (2014), which may have consequences for the time course of linguistic structuring.

Finally, our task could be used as a tool for studying the cultural evolution of sign languages, within the iterated learning paradigm of Kirby and colleagues (Kirby et al., 2014; Motamedi et al., 2017a). In the words of Motamedi et al. (2017b, p. 35), we have investigated in the current study how individuals “improvise solutions to communicative challenges,” but we have not yet looked at the next stages in the process of cultural evolution, which are “how groups of individuals create conventions through interaction and how these conventions are transmitted over time through learning.” Given the likelihood that sign languages originated as gesture without speech (e.g., Senghas and Coppola, 2001; Sandler et al., 2005), our task is appropriate for determining a possible gestural inventory available to deaf people when sign languages emerge.

## Concluding remarks

To understand the nature of second-language learning, it is essential to know what resources learners bring to the task. For adult learners of sign, these resources presumably include manual gesture. Our study aimed to uncover what this manual gesture looks like by eliciting silent gesture with a controlled set of stimuli that in signers elicits entity classifier constructions. We have shown that when sign-naïve adults are required to use silent gesture to describe the locations/orientations of objects, they exhibit a rich repertoire of handshapes. Furthermore, they do not use these handshapes consistently. In contrast, signers have an entity classifier system that is limited to a small set of handshapes which is used in a consistent way. The set of handshapes available to gesturers includes some handshapes that can be used as classifiers in the language they are learning and some that cannot. Therefore, our findings suggest that the challenge for learners of sign is to narrow down their large repertoire to the conventionalized system of the particular language they are learning. It remains to be investigated exactly how learners respond to this challenge.

## Author contributions

Both authors contributed equally to the work reported in this paper and to the writing of the paper.

### Conflict of interest statement

The authors declare that the research was conducted in the absence of any commercial or financial relationships that could be construed as a potential conflict of interest.

## Footnotes

^1^GCSEs (General Certificate of Secondary Education) superseded O-Levels in 1986. The GCSE assessment is taken by students in England and Wales when they are aged 16. It equates roughly with A2 in the Common European Framework of Reference for languages, with students being able to:

- Understand sentences and frequently used expressions related to areas of most immediate relevance.
- Communicate in simple and routine tasks requiring a simple and direct exchange of information on familiar and routine matters
- Describe in simple terms aspects of their background, immediate environment and matters in areas of immediate need.

^2^Gesturers were asked to explain, using only their hands and no voice, how the two pictures differed from each other, whereas signers were asked to describe the differences using BSL.
