# Supplementary material for: Using the Hands to Represent Objects in Space: Gesture as a Substrate for Signed Language Acquisition
Source: Front Psychol. 2017 Nov 20;8:2007. doi: 10.3389/fpsyg.2017.02007 (PMC5715371; doi:10.3389/fpsyg.2017.02007)
Supplement: Supplementary file 1 [file DataSheet1.pdf]

Appendix. Handshapes used by sign-naïve gesturers.

**FULLY CLOSED**			
IN BSL INVENTORY		*NOT IN BSL INVENTORY*	
1. 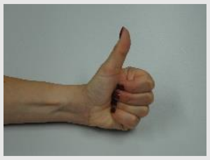	2. 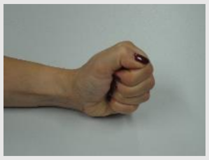	5.* 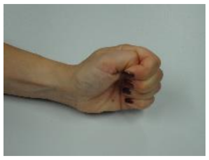	
3. 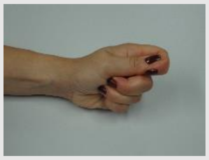	4. 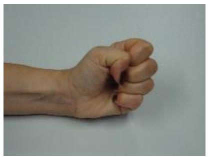		
**CURVED OR BENT**			
IN BSL INVENTORY		*NOT IN BSL INVENTORY*	
6. 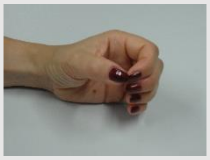	7. 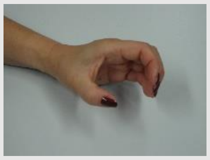	11. 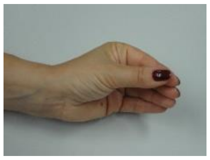	12. 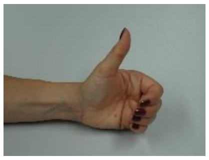
8.* 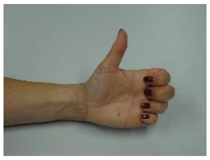	9.* 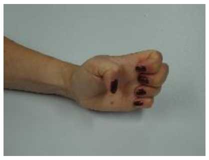	13.* 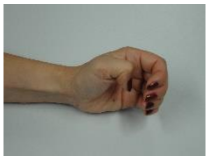	14. 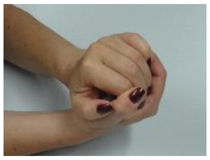
10. 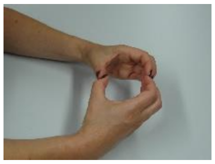			
**FINGERS TOGETHER**			
IN BSL INVENTORY		*NOT IN BSL INVENTORY*	

15. 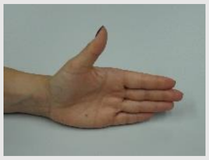	16. 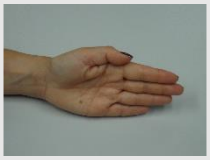		
17. 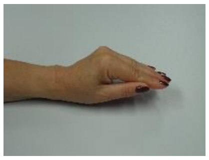	18. 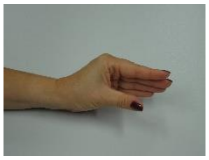		
19. 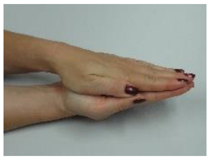			
**FINGERS SPREAD**			
IN BSL INVENTORY		*NOT IN BSL INVENTORY*	
20. 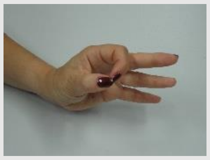	21. 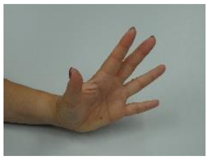		
22. 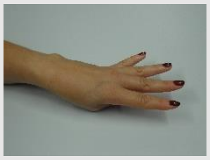	23. 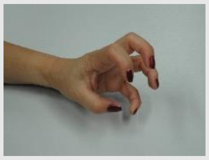		
24. * 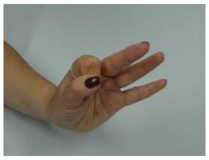	25.* 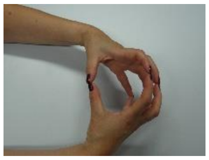		
**FINGERS EXTENDED FROM CLOSED FIST**			
IN BSL INVENTORY		*NOT IN BSL INVENTORY*	

26. 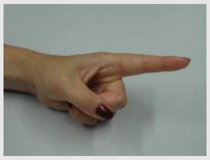	27. 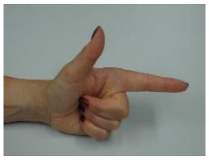	43.* 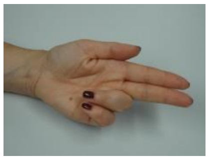	44.* 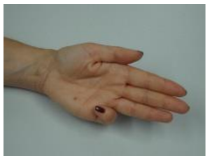
28. 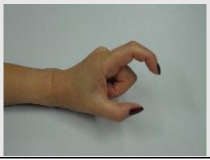	29. 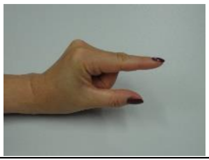		
30. 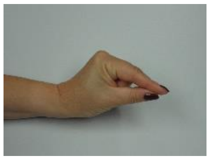	31. 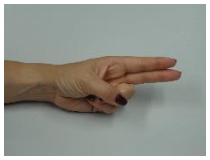		
32. 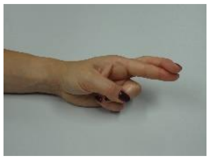	33. 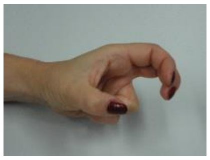		
34. 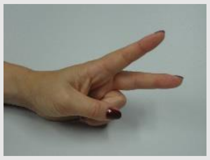	35. 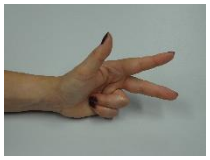		
36. 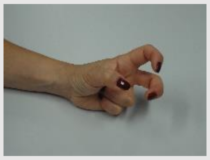	37. 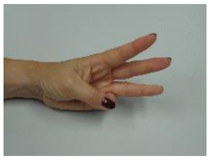		
38.* 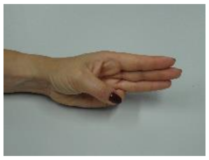	39. 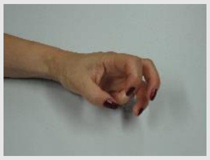		

40. 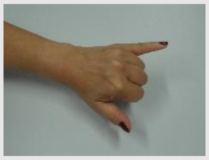	41. 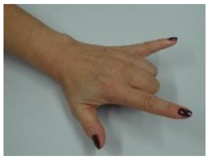		
42. 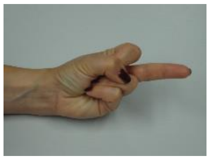			
**MISCELLANEOUS (NOT STRAIGHTFORWARDLY CLASSIFIABLE)**			
45.* 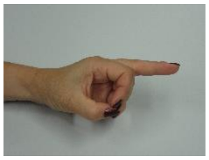	46.* 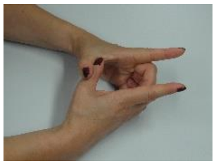	47.** 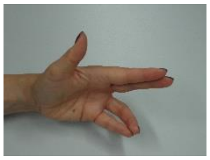	48.* 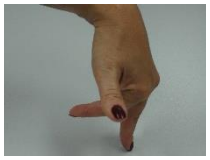
49.** 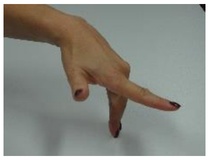	50.* 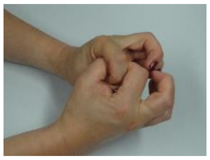	51. 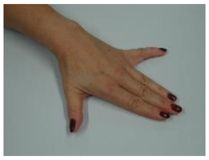	52.** 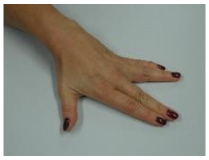
53. * 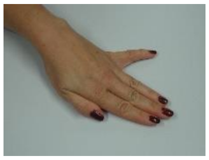			

Key:

Handshape also used by native BSL signers in the classifier elicitation task

* Handshape used by only one participant on only one trial

** Handshape used by only one participant, but on two or more trials
